# 3 Tesla multiparametric MRI for GTV-definition of Dominant Intraprostatic Lesions in patients with Prostate Cancer – an interobserver variability study

**DOI:** 10.1186/1748-717X-8-183

**Published:** 2013-07-22

**Authors:** Hans Christian Rischke, Ursula Nestle, Tobias Fechter, Christian Doll, Natalja Volegova-Neher, Karl Henne, Jutta Scholber, Stefan Knippen, Simon Kirste, Anca L Grosu, Cordula A Jilg

**Affiliations:** 1 Department of Radiation Oncology, University of Freiburg, Robert Koch Str. 3, 79106 Freiburg, Germany; 2 Department of Nuclear Medicine, University of Freiburg, Hugstetter Strasse 55, 79106 Freiburg, Germany; 3 Department of Urology, University of Freiburg, Hugstetter Strasse 55, 79106 Freiburg, Germany

**Keywords:** Prostate cancer, Gross tumor volume, Focal dose escalation, Simultaneous integrated boost, 3 Tesla MRI, Interobserver variability

## Abstract

**Purpose:**

To evaluate the interobserver variability of gross tumor volume (GTV) - delineation of Dominant Intraprostatic Lesions (DIPL) in patients with prostate cancer using published MRI criteria for multiparametric MRI at 3 Tesla by 6 different observers.

**Material and methods:**

90 GTV-datasets based on 15 multiparametric MRI sequences (T2w, diffusion weighted (DWI) and dynamic contrast enhanced (DCE)) of 5 patients with prostate cancer were generated for GTV-delineation of DIPL by 6 observers. The reference GTV-dataset was contoured by a radiologist with expertise in diagnostic imaging of prostate cancer using MRI. Subsequent GTV-delineation was performed by 5 radiation oncologists who received teaching of MRI-features of primary prostate cancer before starting contouring session. GTV-datasets were contoured using Oncentra Masterplan® and iplan® Net. For purposes of comparison GTV-datasets were imported to the Artiview® platform (Aquilab®), GTV-values and the similarity indices or Kappa indices (KI) were calculated with the postulation that a KI > 0.7 indicates excellent, a KI > 0.6 to < 0.7 substantial and KI > 0.5 to < 0.6 moderate agreement. Additionally all observers rated difficulties of contouring for each MRI-sequence using a 3 point rating scale (1 = easy to delineate, 2 = minor difficulties, 3 = major difficulties).

**Results:**

GTV contouring using T2w (KI-T2w = 0.61) and DCE images (KI-DCE = 0.63) resulted in substantial agreement. GTV contouring using DWI images resulted in moderate agreement (KI-DWI = 0.51). KI-T2w and KI-DCE was significantly higher than KI-DWI (p = 0.01 and p = 0.003). Degree of difficulty in contouring GTV was significantly lower using T2w and DCE compared to DWI-sequences (both p < 0.0001). Analysis of delineation differences revealed inadequate comparison of functional (DWI, DCE) to anatomical sequences (T2w) and lack of awareness of non-specific imaging findings as a source of erroneous delineation.

**Conclusions:**

Using T2w and DCE sequences at 3 Tesla for GTV-definition of DIPL in prostate cancer patients by radiation oncologists with knowledge of MRI features results in substantial agreement compared to an experienced MRI-radiologist, but for radiotherapy purposes higher KI are desirable, strengthen the need for expert surveillance. DWI sequence for GTV delineation was considered as difficult in application.

## Introduction

Radiotherapy (RT) of primary prostate cancer (PCa) has been modified in the past decade by using image-guided radiotherapy (IGRT) and intensity modulated radiotherapy (IMRT) techniques [1]. Whole gland dose escalation with IMRT proved to be safe in respect of acute and late toxicities [2-4]. Although prostate cancer is typically a multifocal disease, histopathologic studies revealed that most patients with prostate cancer have at least one or two dominant intraprostatic tumor lesions (DIPL) [5,6]. For patients scheduled for primary radical radiotherapy obtaining high irradiation doses of the whole prostate are crucial to achieve high biochemical and clinical control rates [7]. However the risk of toxicity, especially in the rectal mucosa inevitably increases with dose escalatation [8], thus requiring highly precise and accurate radiation techniques. There is evidence that local prostate cancer recurrence after primary radiotherapy develops from the origination of the primary tumor or from the initial dominant intraprostatic tumor burden [9,10]. Experience with IMRT has led to the concept of focal dose-escalation using simultaneous integrated boost of DIPL. Local dose escalation on DIPL may result in significant improved disease control without increasing normal tissue complication probability (mainly acute and chronic rectal mucositis/proctitis). This therapeutic approach has been calculated by Niyazi et al. in a mathematical model based on different assumptions of responsiveness of prostate cancer to irradiation and different sensitivities and specificities of an appropriate imaging method considering choline PET [11].

Many studies with histopathologic comparison on whole-mount sections as reference standard have shown that Magnetic Resonance Imaging (MRI) using anatomic and functional sequences like Magnetic Resonance Spectroscopy (MRS), Dynamic Contrast Enhanced MRI (DCE-MRI) and Diffusion weighted Imaging (DWI) results in high accuracies in detecting primary prostate cancer due to excellent spatial resolution with clear depiction of anatomy/pathoanatomy in combination with visualization of functional properties of prostatic lesions [12-23]. DWI-MRI in conjunction with T2-weighted showed accuracies of 81% and 89% at 1.5 Tesla respectively [17,18]. DCE-MRI showed a sensitivity and specificity for identification of cancer foci > 0.5 mL of 86% and 94%, respectively [19]. Furthermore a combination of two functional sequences at 1.5 Tesla resulted in a significantly improved area under the receiver operating characteristic (ROC) curve compared to a single functional parameter when whole-mount sections with histologically defined tumor outlines were used as reference standard. Using the combination of apparent diffusion coefficient and initial area under the gadolinium plasma concentration-time curve for detection of cancer foci resulted in an area under the ROC curve of 0.94 reflecting high accuracy. Combination of all three functional parameters (DWI, DCE-MRI and MRS) showed no further improvement [20]. Using T2w sequences at 3 Tesla results in reported sensitivities and specificities of 80%–88% and 96%–100%, respectively [24]. Prostate imaging at 3 Tesla benefits from higher signal to noise ratio (SNR), enables higher quality imaging than obtained at 1.5 Tesla and moreover the use of an endorectal coil can be obviated with satisfying image quality [25] and without distortion of pelvic anatomy which is important for radiotherapy planning [26]. Recently the European Society of Urogenital Radiology (ESUR) published MR guidelines for imaging in prostate cancer and structured reporting [27].

MRI-Criteria to identify an intraprostatic tumor lesion are different throughout the MRI-sequences [27]. Few studies based on consensus reading of a radiologist and radiation oncologist using functional MRI sequences for definition of DIPL have shown that focal dose escalation results in low acute toxicities [28,29] with better sparing of the rectal wall [30].

We wondered if knowledge and application of MRI-criteria (Table 1, that are close to the recent published ESUR-criteria) of DIPL leads to identical GTV-definitions by different radiation-oncologists in comparison to a radiologist with special knowledge of prostate-MRI. Therefore the aims of the study were threefold: first to analyze the practicability of MRI-criteria that can be used to define a DIPL in 3 Tesla MRI-sequences, second to evaluate the interobserver variability of radiation-oncologists versus an experienced radiologist and third to evaluate possible reasons of increased interobserver-variabilities.

**Table 1:** Description of MRI criteria suggestive for malignancy or DIPL according to different MRI sequences

**MRI sequence**	**MRI criteria suggestive for malignancy or DIPL**
**T2w**	Peripheral zone: inhomogeneous, irregular, low-signal intensity lesion with unclear margins or diffuse extension and mass effect.
	Central gland (transition zone), [23] homogeneous low-signal intensity region with:
	• Poorly defined or spiculated lesion margins
	• Lack of a low-signal-intensity rim (seen commonly in association with benign adenomatous nodules),
	• Interruption of the surgical pseudocapsule (transition zone–to–peripheral zone boundary of low signal intensity),
	• Urethral or anterior fibromuscular stromal invasion, and
	• Lenticular shape
**Diffusion weighed (DWI, using ADC-maps)**	Round-ellipsoid low intensity regions are suggestive of prostate cancer lesions [21,22].
	Potential limitation: the high prevalence of benign prostate hyperplasia (BPH) may lead to low intensity nodules like cancerous tissue.
**Dynamic contrast enhanced (DCE)**	Focus of asymmetric, early and intense enhancement with rapid washout compared to the background.
	Potential limitation: enhancing prostatitis in the peripheral zone and enhancing BPH in the transition zone [32].

## Methods

### Patients

Patients referred for irradiation of histopathologic proven primary prostate cancer and who received pre-therapeutic multiparametric 3 Tesla MRI with MRI-identifiable prostatic lesions that suggest malignancy according to the MRI-criteria (Table 1) were selected from our database. For this retrospective study, the University of Freiburg Institutional Review Board waived the consent requirements. Patient characteristics were as follows:

1. Pat. No. 1, 73 years, cT2b cN0 M0, Gleason 3 + 4, initial PSA 14.6 ng/mL

2. Pat. No. 2, 80 years, cT3b cN0 M0, Gleason 4 + 5, initial PSA 10.4 ng/mL

3. Pat. No. 3, 63 years, cT2c cN0 M0, Gleason 3 + 4, initial PSA 5.1 ng/mL

4. Pat. No. 4, 69 years, pT3b cN0 M0, Gleason 4 + 3, initial PSA 9.1 ng/mL

5. Pat. No. 5, 71 years, cT3a cN0 M0, Gleason 3 + 4, initial PSA 9.4 ng/mL

### MRI Technique

All MRI scans were acquired on a 3 Tesla system (Trio Tim, Siemens Medical Solutions, Erlangen, Germany), equipped with surface phased array (Body Matrix, Siemens Medical Solutions). Imaging was performed by the following sequences:

T2-weighted turbo spin echo (TSE) sequences in the axial, sagittal and coronal planes (repetition time [TR], 8000 ms; echo time [TE], 110 ms; flip angle 130; field of view 170 × 170 mm; thickness 3 mm; section gap 0.3; matrix, 256 × 256).

T1-weighted (TSE) series of the whole pelvis was then obtained with the following parameters: repetition time [TR], 816 ms; echo time [TE], 11 ms; flip angle 140; field of view 380 × 300 mm; thickness 3 mm; section gap 0.3; matrix, 384 × 306.

DWI-sequence had repetition time [TR], 3100 ms; echo time [TE], 85 ms; b-factor 1000; field of view 220 × 220 mm; thickness 3 mm; section gap 0.3; matrix, 124 × 124.

The last series performed was a 3D, fast low-angle shot (FLASH), T1-weighted spoiled gradient-echo sequence in axial plane (TR, 3,96 ms; TE, 1.38 ms; flip angle 12,33, field of view 340 × 265 mm, thickness 1.65 mm; section gap 0; matrix, 384 × 300) to perform measurements in rapid succession, immediately following completion of an intravenous bolus injection of 0.1 ml/kg gadopentetate dimeglumine (Multihance, Bracco) using a power injector (Medtron) at 3 ml/s followed by a 30 ml saline flush, 54 contrast-enhanced sets of images were acquired sequentially without a delay between acquisitions, therefore time resolution was 7 seconds.

### Image analysis

Dicom datasets of T2w, DWI and DCE MRI-sequences of each patient were imported into a RT-planning system (Oncentra Masterplan® or iplan® Net), that is used in daily routine ensuring familiarity with the delineation process. Slices from the DCE-image-series with visually determinated early peak enhancement in suspicious lesions, appropriate for delineation, were preselected by the expert radiologist before import into the RT-planning system.

All radiation oncologists, familiar with delineation of the prostate as whole organ using MRI scans, attended two 1h-teaching lectures, in which prostate anatomy and biophysical principles of anatomic and functional MRI-sequences (Table 2, [27,31-40]) and the published criteria suggestive for malignancy (Table 1, [21-23,32]) were demonstrated and explained by a radiologist with 8 years of experience of urogenital radiologic imaging especially prostate MRI. Interactive discussions of case studies taken from the literature and from the own department was an integral part of the lecture.

**Table 2:** Description of biophysical principles and image characteristics of different MRI sequences

**MRI sequence**	**Biophysical principles and image characteristics**
**T2w**	*Normal* peripheral zone contains relatively high proton density, leading to a homogeneous high signal intensity. Linear, wedge-shaped, or oval low-signal intensity lesions may be present but are considered non-malignant [31].
	*Normal* central gland: variable amounts of intermediate signal intensity, which is often replaced by well-circumscribed hyperplastic nodules of BPH with variable signal intensity [32-34].
**Diffusion weighed (DWI)**	DWI-MRI measures the Brownian motion of water molecules. Reduced diffusion of water in prostate cancer is attributed to the increased cellularity of malignant lesions, with reduction of the extracellular space and restriction of the motion of a larger portion of water molecules to the intracellular space [35]. The amount of diffusion in tissue is determined quantitatively by the apparent diffusion coefficient (ADC) [32]. Lower ADC values present malignant lesions encoded by lower signal intensity (similar to T2w images). Similar to T2w-images detection of prostate cancer in the peripheral zone is more accurate than in the transitional zone, where the high prevalence of benign prostate hyperplasia (BPH) may lead to reduced ADC values like cancerous tissue. Higher Gleason score is associated with decreased ADC, likely due to the dedifferentiated infiltrative growth of these tumors [36,37]. DWI-image quality and contrast resolution may be hampered by tumor characteristics (e.g. low to intermediate Gleason score), susceptibility artefacts due to magnetic field inhomogeneity and reduced in-plane resolution [27,32].
**Dynamic contrast enhanced (DCE)**	DCE-MRI can visualize increased tumor vascularity in prostate cancer lesions. Prostate cancer lesions typically demonstrate early, rapid and intense enhancement with quick wash-out [38,39]. Tumor vessels are different from physiological vessels and typically have a higher permeability leading to contrast agent leakage in the extracellular-extravascular space. DCE MR images need to be evaluated by direct visual interpretation of dynamic enhanced T1-weighted images. Potential limitations of DCE-MRI are that it may not reliably differentiate prostatitis in the peripheral zone and transition zone tumors from BPH [32]. Second signal intensity may be analysed using time-signal parameters, e.g. determining peak enhancement or calculation of initial area under the curve in the first 60 seconds (iAUC60) [40].

**Figure 1:**
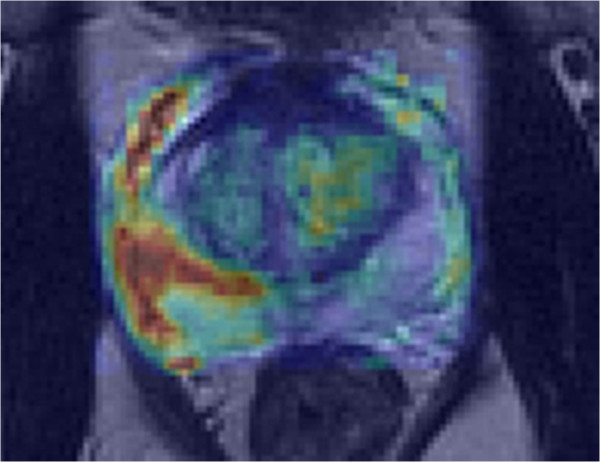
**Patient No. 4.** iAUC60 values are converted into pseudocolor parametric maps and overlaid to the anatomic T2w images to support reading of the DCE-images.

In addition to visual analyses of DCE-MRI images calculated iAUC60 values [40] were converted into pseudocolour parametric maps and overlaid to the anatomic T2-weighted images to support reading of the DCE-images (example, see Figure 1).

The contouring radiation oncologist had access to the MRI-report and the clinical staging parameters for each of the five selected patients with histopathologically (biopsy) verified prostate cancer. All radiation oncologists were equipped with a hand-out containing a summary of the above listed delineation criteria for the different sequences (Table 1) and an atlas of typical pathologic findings available at hand when performing delineation on their own. First the GTV1 was contoured on the T2w images (violet colour), second GTV2 was contoured on the DWI-images (red colour) and third GTV3 was delineated in the DCE-image-series (yellow colour) by each radiation oncologist (observer) and the expert radiologist (reference-dataset). Using the Oncentra Masterplan® of iplan® Net fusion tool the T2w images were permanently underlaid to the functional sequences (DWI, DCE) with user enabled variable opacity for proper visualization of the organ borders. Once the delineation process of GTV 1 to GTV 3 was started it was accomplished in one session for each patient. Observers were instructed not to compare DWI vs. DCE contours as an aim of the study was not to generate a consensus volume but to evaluate how each functional sequence is suitable for application of MRI-criteria by a radiation oncologist. However observers were instructed to compare functional to anatomical T2w sequence with respect to anatomy and organ borders. Each observer rated difficulties of contouring according to the used MRI-sequence using a 3-point scale rating scale (1 = easy to delineate, 2 = minor difficulties, 3 = major difficulties). Finally 90 GTV-datasets with definition of DIPL based on 5 patients, each examined with 3 different MRI-sequences were generated by 6 observers.

### Statistics

For comparison purposes GTV datasets were imported to the Artiview® platform (Aquilab®). Using Artiview®-Software-package GTV-volumes and Kappa indices (KI) were calculated. Kappa statistic is currently the standard to analyze reproducibility between to observers based on binary questions [41] including digital (pixel based) imaging [42]. Kappa-index reflects agreement on pixel-by-pixel basis with chance correction. It is defined by Kappa = Po - Pc/1 – Pc, in which Po is the observed percentage of agreement (the percentage of targets (pixels) rated the same by different observers) and Pc is the percent of agreement that would occur by chance alone [42]. According to the study it describes the ratio between the intersection of the delineated volume for a given observer x with its corresponding reference volume and their average. A Kappa-value of 1 indicates perfect agreement, Kappa = 0 indicates agreement equal to pure chance. It is generally accepted that Kappa > 0.7 indicates excellent agreement [42], although others suggested interpretation of kappa-values from 0.41 to 0.6 as moderate, from 0.61 to 0.8 as substantial and > 0.81 as excellent [43]. All contours were reviewed to analyze reasons for differences in GTV delineation. Statistical analysis was done by Mann–Whitney-Test with a significance level at 0.05 (IBM-SPSS-STATISTICS-Version19 Software).

## Results

**Figure 2:**
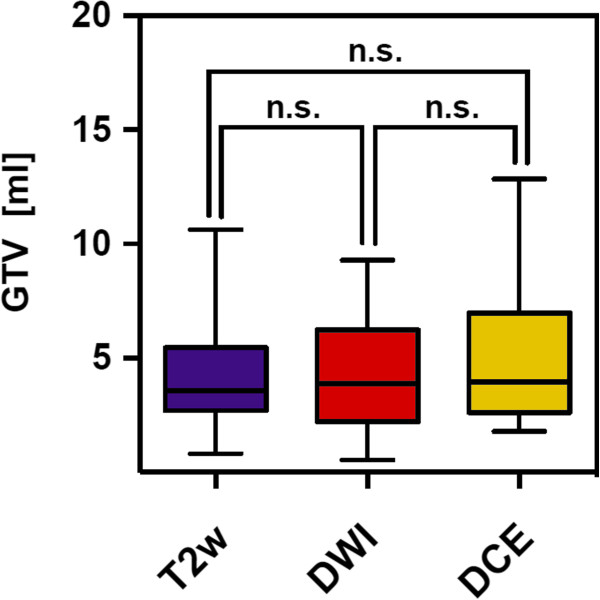
**Box plots with median, standard deviation and range values of the GTV contoured by 6 observers (including reference observer) upon three different MRI-sequences for all patients.** There were no statistical difference between the different GTV results for each sequence and for all patients together (n.s. = not significant).

**Figure 3:**
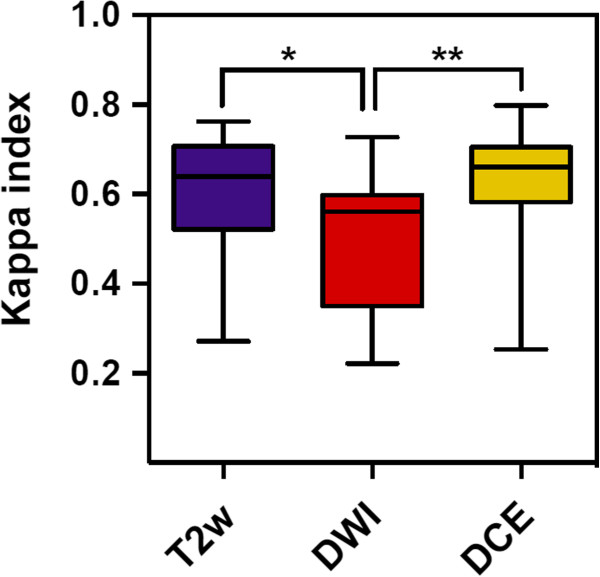
KI-T2w and KI-DCE was significantly higher than KI-DWI, *p = 0.01 (CI: 0.02-0.18) and **p = 0.0027 (CI: 0.2-0.04), respectively.

**Figure 4:**
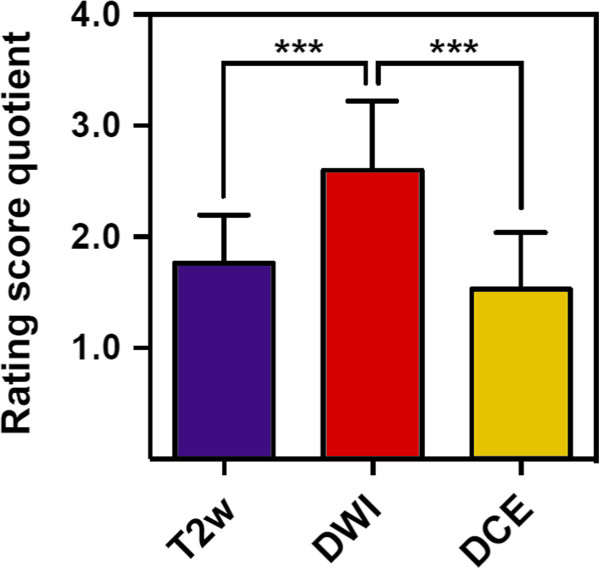
**Rating score Quotient at T2w, DCE and DWI.** Degree of difficulty in contouring GTV was significantly lower using T2w and DCE compared to DWI-sequences, ***p < 0.0001 (CI: 1.11-0.56) respectively ***p < 0.001 (CI:0.77-1.36).

Different MRI-sequences lead to different GTV created by 6 observers within the same patient. Data on different GTV results are given in Tables 3 and 4, there were no statistical difference between the different GTV results for each sequence and for all patients together (Table 4, Figure 2). The Kappa-indices throughout the three different MRI-sequences are listed in Table 5. At T2w a KI > 0.6 had been obtained in 15 of 25 GTV-definitions indicating substantial to excellent agreement in 60%. At DWI a KI > 0.6 had been obtained in 6 of 25 GTV-definitions, indicating substantial to excellent agreement in 24%. At DCE a KI > 0.6 had been obtained in 18 of 25 GTV-definitions indicating substantial to excellent agreement in 72% of contoured GTV with the reference contour. Mean KI at T2w and DCE was 0.61 (SD: 0.12) and 0.63 (SD: 0.12) respectively. Mean KI at DWI was 0.51 (SD: 0.15). Both KI-T2w and KI-DCE were significantly higher than KI-DWI, p = 0.01 (CI: 0.02-0.18) and p = 0.0027 (CI: 0.2-0.04), respectively (Figure 3). Rating score quotient was at T2w 1.76 (SD: 0.43), at DCE 1.53 (SD:0.51) and at DWI 2.6 (SD: 0.62). The degree of difficulty in contouring GTV was significantly lower using T2w and DCE compared to DWI-sequences, p < 0.0001 (CI: 1.11-0.56) respectively p < 0.001 (CI:0.77-1.36) (Figure 4).

**Table 3:** Median, mean and standard deviation values of the GTV contoured by 6 observers (including reference observer) upon three different MRI-sequences for each patient

	**T2w [ml]**			**DWI [ml]**			**DCE [ml]**		
	**Median**	**Mean**	**SD**	**Median**	**Mean**	**SD**	**Median**	**Mean**	**SD**
Pat. No1	3.7	4.1	1.5	4.0	3.8	1.6	3.3	4.7	4.2
Pat. No2	6.7	6.8	2.5	8.1	7.0	2.8	7.3	8.4	3.1
Pat. No3	2.0	2.1	0.5	1.8	2.6	2.5	2.3	2.7	0.9
Pat. No4	4.7	5.2	1.7	5.2	5.3	1.9	6.8	7.1	3.1
Pat. No5	3.4	3.2	1.3	2.9	3.0	0.9	2.8	3.4	1.1

**Table 4:** Shows different GTV summarized for all patients

**GTV-Volumina**	**T2w [ml]**	**DWI [ml]**	**DCE [ml]**
**Mean**	4.3	4.4	5.3
**Median**	3.6	3.9	4.0
**SD**	2.3	2.5	3.4

**Table 5:** Kappa-indices throughout the different MRI-sequences

	**Pat. No. 1**	**Pat. No. 2**	**Pat. No. 3**	**Pat. No. 4**	**Pat. No. 5**
**T2w**	Kappa index	Kappa index	Kappa index	Kappa index	Kappa index
Reference observer	1	1	1	1	1
Observer A	**0,639**	0,469	0,532	0,496	**0,671**
Observer B	**0,736**	**0,695**	**0,724**	0,471	**0,646**
Observer C	**0,720**	0,549	**0,653**	**0,634**	**0,691**
Observer D	**0,652**	**0,762**	0,579	**0,748**	**0,605**
Observer E	**0,759**	0,511	0,539	0,475	0,272
T2w Rating-score quotient	**1,67**	**1,83**	**1,67**	**1,83**	**1,83**
**Diffusion weighed (DWI)**	Kappa index	Kappa index	Kappa index	Kappa index	Kappa index
Reference observer	1	1	1	1	1
Observer A	0,561	0,315	0,222	0,593	**0,603**
Observer B	**0,727**	0,528	0,304	**0,648**	0,497
Observer C	0,582	0,568	0,337	0,477	0,563
Observer D	0,586	**0,687**	0,358	0,588	0,555
Observer E	**0,692**	0,470	0,237	0,343	**0,672**
DWI Rating-score quotient	**2,17**	**2,83**	**3**	**2,17**	**2,83**
**Dynamic contrast enhanced (DCE)**	Kappa index	Kappa index	Kappa index	Kappa index	Kappa index
Reference observer	1	1	1	1	1
Observer A	0,254	**0,641**	**0,669**	0,471	0,563
Observer B	**0,737**	**0,653**	**0,640**	**0,717**	**0,687**
Observer C	**0,710**	**0,691**	**0,651**	**0,775**	0,409
Observer D	0,476	**0,651**	**0,746**	**0,798**	**0,679**
Observer E	0,000	**0,677**	**0,691**	**0,643**	0,543
DCE Rating-score quotient	**1,67**	**1,5**	**1,33**	**1,33**	**1,83**

KI = Kappa-Index. KI-values > 0.6 are bold and regarded as substantial agreement respectively. KI-values > 0.7 indicate excellent agreement with the reference volume per definition (please methods section). Rating score quotient is the average of rating points assigned by all observers (including reference) (1 = easy to delineate, 2 = minor difficulties, 3 = major difficulties).

**Figure 5:**
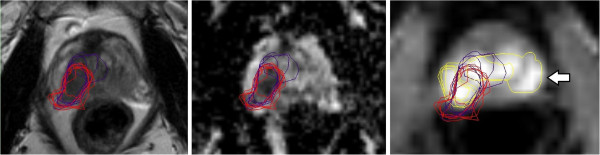
**(Patient No.1).** Transversal slices through the middle prostate gland; left = T2w-, middle = DWI-, right = DCE-sequence. Left and middle picture show T2w (violet contour) and DWI (red contour) based GTV-delineation respectively. On the right image additionally DCE (yellow contour) based GTV-delineation; the arrow indicates delineation of non-specific enhancement by two observers in the left gland symmetric to the right side.

**Figure 6:**
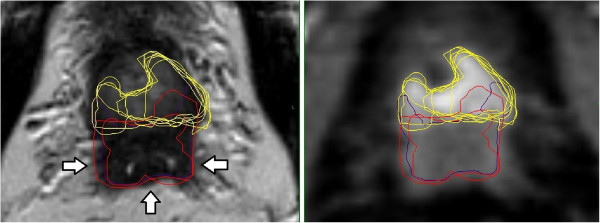
**(Patient No. 2).** Transversal slices through prostate base and adjacent seminal vesicles; left = T2w-, right = DCE-sequence. Left: the diffuse low signal intensity forced two observers to include the junction of seminal vesicles in the prostate base (violet and red contours, arrows) into the GTV at T2w images. Right: DCE based GTV-definition (yellow contours).

**Figure 7:**
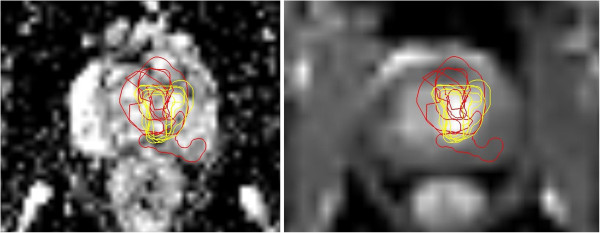
**(Patient No. 3).** Transversal slices through the middle of the prostate; left = DWI-, right = DCE-sequence. Left: DWI based GTV-delineation (red contours). Right: DCE based GTV-delineation (yellow contours). T2w-GTV-contours are not depicted for better survey.

**Figure 8:**
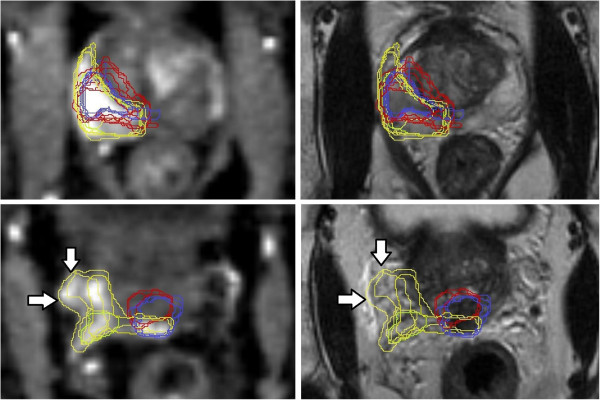
**(Patient No. 4).** Transversal slices in the middle third (above) and near the prostate base (below) of the prostate gland; above/below left = DCE-, above/below right = T2w-sequence. Above left and right: DCE based GTV-delineation (yellow contours) with high KI. Below left: two observers erroneously delineated laterally adjacent enhancing periprostatic vascular structures near the prostate base (arrows indicating yellow contours). Below right: arrows indicate presence of periprostatic vessels.

**Figure 9:**
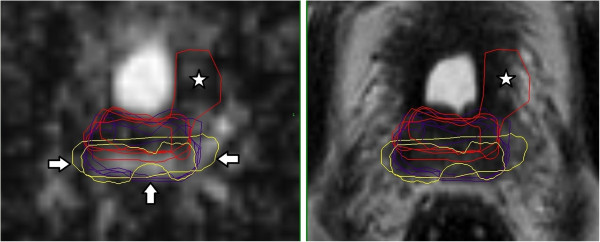
**(Patient No. 5).** Transversal slices through near the prostate base; left = DWI-, right = T2w-sequence. DWI based GTV-delineation (red contours) does not correspond to T2w based GTV-delineation (violet contours). Asterisk marks the erroneously contoured area in both pictures. Additionally erroneous delineation of unremarkable seminal vesicles (arrows in the left picture).

All contours were reviewed to analyze reasons for differences in GTV delineation. Figure 5 (Patient No. 1) shows that T2w-GTV-delineation resulted in high KI compared to DCE-GTV-delineation (Table 5). The reason for this was widely distributed symmetric contrast enhancement in both prostate lobes (Figure 5, arrow in the right picture) causing confusion concerning DIPL borders. Symmetric enhancement at DCE without corresponding criteria of DIPL in other sequences (including T2w as required by the delineation-instructions) should raise suspicion of the presence of rather benign changes such as prostatitis in the peripheral zone or BPH in the transition zone [32]. Figure 6 (Patient No. 2) demonstrates the reason for a significant different delineation contour owed to inadequately comparison of the sequences to each other. Figure 6, left picture, shows a transversal slice through prostate base and adjacent seminal vesicles, the diffuse low signal intensity forced two observers to include the junction of seminal vesicles in the prostate base (red contours) into the GTV at T2w and DWI images (latter not shown). This was based on the assumption that this low signal intensity is suggestive of malignancy but no enhancement can be seen at DCE and the prostate base should not be considered to be infiltrated by the enhancing DIPL (Figure 6, right picture). Figure 7 (Patient No. 3) shows the difference between DWI and DCE used for GTV-delineation; DWI-GTV-delineation (red contours) resulted in a high interobserver variability/low KI compared to DCE-GTV-delineation, where all observers including reference agreed substantially using DCE images (Figure 7, right). Figure 8 (Patient No. 4), above left and right, shows an example of excellent KI of DCE-GTV-delineation. Figure 8 (below left and right) two observers erroneously delineated laterally adjacent periprostatic vascular structures. Figure 9 (Patient No. 5) a delineation contour exceeds the organ contour of the prostate gland by far as one observer who failed to correlate the DWI image (Figure 9, left) with the corresponding T2w image (Figure 9, right). Figure 9 also demonstrates that if all observers would have had performed comparison with the anatomic conditions no GTV would contain the high signal intense area (arrows in Figure 9, left), that is equivalent to unremarkable seminal vesicles at the base of prostate gland.

## Discussion

The strategy of focal dose escalation to DIPL within the prostate to improve local tumor control and outcome of primarily irradiated prostate cancer patients has gained increasing interest in the past decade [11,28-30,44]. A large multicentre randomized trial has been initiated that compares focal dose escalation based on multiparametric MRI findings vs. standard whole gland irradiation. In this trial GTV-delineation is performed by experts in the field of multiparametric prostate-MRI [45].

Other ongoing trials also use MRI to define the GTV for focal dose escalation (e.g. ‘Tumor TARGET Prostate Cancer’ (NCT01802242) or ‘The HEIGHT Trial’ (NCT01411332)). Many studies using a combination of anatomic with functional MRI sequences for detection of DIPL having whole-mount histopathologic as reference resulted in the definition of MRI guidelines by an expert panel [27]. However published anatomic and functional MRI criteria for DIPL have not yet been used in terms of GTV-delineation by different radiation oncologists to elucidate feasibility and potential confounding factors throughout application in clinical practice. To the best of our knowledge this is the first study that compares interobserver variability using multiparametric MRI for GTV-Definition of DIPL in patients with prostate cancer. The GTV-volumes were similar throughout the different MRI-sequences, although increased standard deviations indicate delineation difficulties in some sequences (Tables 3 and 4, Figure 2). We were able to show that a comprehensive but tailored teaching of radiation oncologists about published and widely accepted MRI criteria of DIPL results in substantial to partially excellent agreement compared to an experienced prostate MRI reader depending on the used MRI-sequences (Table 5). Mean KI at T2w and DCE was significantly higher than KI-DWI (Figure 3). Additionally we measured applicability with a 3-point rating score describing difficulties of the delineation process. We found that the degree of difficulty in contouring the GTV was significantly lower using T2w and DCE compared to DWI-sequences (p < 0.0001 for both, Figure 4).

We highlight some important aspects of the delineation process. First, it is important to have anatomic details provided by the T2w-sequence as an underlying dataset to fuse with the functional dataset (DWI, DCE). Complementary morphologic information is essential to avoid delineation errors exceeding organ contours like those that are described in Figures 8 and 9. Second, different signal characteristics of functional sequences should be critically compared to each other to check for possible non-specific findings like bilateral symmetric contrast enhancement described in Figure 5. Furthermore one has to keep in mind that the specificity of functional MRI-sequences is higher than the anatomic T2w sequences [12-23], but depends not only on the signal characteristic but also on the signal distribution in context with the surrounding anatomy [21,22,32]. Inadequate comparison may lead to delineation errors as described in Figures 6 and 9. T2w-sequences have a lower specificity for tumor detection than DWI or DCE-sequences [46-48]. However GTV-delineations done by DWI and DCE-MRI sometimes may not co-localize well in tumor-bearing prostate glands because both parameters reflect different tissue properties that are associated with the presence of tumor. To manage this problem Groenendal et al. suggested if DWI and DCE give consistent information, the delineation of a target can be straightforward, because there is a high probability that regions identified by both modalities contain tumor tissue. When the two imaging modalities give inconsistent information, the probability that tumor is present is smaller. A practical approach could be to treat the voxels on which the two modalities agree as the GTV. In case only one of the two modalities indicates a voxel as suspicious, the region could be considered a ‘high-risk CTV’. One could choose not to boost these regions, but in any case safe margins should be applied around these regions [49].

Our study has some limitations. We selected 5 consecutive patients that received functional MRI at 3 Tesla from our database with clearly visible DIPL. Depending on the type of cancer, its growth pattern and patient specific conditions (e.g. antiandrogen therapy prior to MRI [50]) visualization of DIPL may be hampered by difficulties to distinguish or by lacking distinct lesions [28]. 3 Tesla functional MRI is currently the imaging device with the highest accuracy in detection of DIPL due to different functional sequences offering important additional information about specific tissue characteristics. Magnetic resonance spectroscopy (MRS) – sequences were not available, adding MRS-sequences would have led to 15 min extra examination time and is not part of the routine diagnostic work up in our radiology department. Knowing the reported high specificity (but low sensitivity) of MRS to characterize prostate cancer nodules [51] and the limited spatial resolution, we prefer image characteristics of the two other functional MRI-sequences (DCE, DWI) for GTV-delineation and comparison metrics. However preselection of DCE-image-series, of iAUC60-derived maps and the ADC-maps by the reference radiologist may have introduced a bias in the image analysis. In fact according to the ESUR-guidelines further analyses of image data, e.g. comparison of ADC-maps with b-value images (at > b800) and generating DCE-enhancement curves in suspicious regions are useful to more precisely characterize image findings. Future studies may use the newly introduced PI-RADS scoring system to describe DIPL. Standard of reference was predetermined by a radiologist with thorough knowledge of imaging features of prostate cancer using functional MRI but we did not have a whole-mounted histopathologic reference standard. Although GTV delineation was performed with caution by the reference radiologist it cannot be ruled out that in the situation of low tumor to background contrast (e.g. Figure 7) the GTV was arbitrarily delineated to some extent and does not necessarily represent the true tumor extension. It is important to emphasize that the major goal in terms of dose escalation is to define the approximate volume of the dominant intraprostatic lesion, which will be irradiated with a certain safety margin that corrects for intrafraction organ movement and therefore submillimeter precision will not necessarily translate in altered planning target volumes (PTV). In fact it is always an individual decision whether dose escalation is feasible taking into account normal tissue dose constraints that may be influenced significantly by individual factors [30].

The teaching lectures and hand-outs (Table 1) comprised all currently available information to perform the required GTV-delineation. Our results do reflect that the attending radiation oncologists did successfully delineate GTV in some cases according to the MRI sequence. However our analysis also show that significant slips of the pen do occur while GTV-delineation in different MRI sequences and comparison to each other is challenging and therefore should not be used in a clinical setting without expert surveillance. Segmentation algorithms may be useful to reduce interobserver variability of prostate organ delineation [52]. In addition Groenendaal et al. described a logistic regression model that predicts tumor presence on a voxel level in the peripheral zone of the prostate gland based on ADC and K-trans values within a voxel. They found a high correspondence of model and pathologic findings at an AUC of 0.89 [53]. From the radiation-oncologists point of view an imaging device that offers objective and reliable detection of DIPL seems strongly desirable. For this purpose the proposed statistic model showing a high diagnostic performance may be a useful tool for the peripheral zone were most of the tumors occur [53].

MRI has been shown to be improve target delineation [54,55] and isotropic voxels reduce delineation discrepancies [56]. But even using established MRI-sequences (T2w) for prostate organ delineation may result in significant variability as was recently shown in a multi-observer, -center and -sequence study based in T2w-sequences [57]. In this study Nyholm et al. found that the imaging sequence appears to have a large influence on the delineation variability. Interestingly they found that images with optimal quality were associated with the largest delineation variability. They concluded that increased amount of information increases the scope of interpretation and hence the importance of training and experience. Our results lead to a similar conclusion that a second observer (experienced radiologist) opinion is required until the skills of functional MRI delineation have been developed and trained by the radiation oncologists. Positron-Emission-Tomography in combination with computed tomography (PET/CT) may offer appropriate visualization of functional properties depending on the radiotracer, but experience with labelled choline in the untreated prostate with presence of PCa showed conflicting results with limited accuracy [58-60]. In this respect new and highly specific radiotracers for prostate cancer imaging are required, that are more appropriate for radiotherapy purposes [61].

## Conclusions

Using T2w and DCE sequences at 3 Tesla for GTV-definition of DIPL in prostate cancer patients by radiation oncologists with knowledge of MRI features results in substantial agreement compared to an experienced MRI-radiologist, but for radiotherapy purposes higher KI are desirable. DWI sequences for GTV delineation were considered as difficult in application and resulted in only moderate interobserver agreement. From the radiation oncologists point of view GTV-delineation in different MRI sequences and comparison to each other is challenging and therefore should not be used in a clinical setting without expert surveillance.

## Competing interest

The authors declare that they have no competing interest.

## Authors’ contributions

HCR and UN carried out the design of the study. HCR, NV, KH, JS, SKn, SKi carried out imaging analyses. HCR, UN carried out the drafting of the manuscript and analysis and interpretation of the data. TF, CD and CAJ participated in the analysis of the data. ALG participated in the methodological design and interpretation of the data. CAJ participated in the analysis and statistics of the data. All authors read and approved the final manuscript.
